# Rheological Behavior of SAE50 Oil–SnO_2_–CeO_2_ Hybrid Nanofluid: Experimental Investigation and Modeling Utilizing Response Surface Method and Machine Learning Techniques

**DOI:** 10.1186/s11671-022-03756-7

**Published:** 2022-12-08

**Authors:** Mojtaba Sepehrnia, Mohammad Lotfalipour, Mahdi Malekiyan, Mahsa Karimi, Somayeh Davoodabadi Farahani

**Affiliations:** 1 Department of Mechanical Engineering, Shahabdanesh University, Qom, Iran; 2 Department of Mechanical Engineering, Technical and Vocational University, Qom, Iran; 3 Faculty of Mechanical Engineering, University of Kashan, Kashan, Iran; 4 School of Mechanical Engineering, Arak University of Technology, Arak, Iran

**Keywords:** Experimental study, Hybrid nanofluid, Cerium oxide–tin oxide, RSM, ANFIS, Machine learning

## Abstract

In this study, for the first time, the effects of temperature and nanopowder volume fraction (NPSVF) on the viscosity and the rheological behavior of SAE50–SnO_2_–CeO_2_ hybrid nanofluid have been studied experimentally. Nanofluids in NPSVFs of 0.25% to 1.5% have been made by a two-step method. Experiments have been performed at temperatures of 25 to 67 °C and shear rates (SRs) of 1333 to 2932.6 s^−1^. The results revealed that for base fluid and nanofluid, shear stress increases with increasing SR and decreasing temperature. By increasing the temperature to about 42 °C at a NPSVF of 1.5%, about 89.36% reduction in viscosity is observed. The viscosity increases with increasing NPSVF about 37.18% at 25 °C. In all states, a non-Newtonian pseudo-plastic behavior has been observed for the base fluid and nanofluid. The highest relative viscosity occurs for NPSVF = 1.5%, temperature = 25 °C and SR = 2932.6 s^−1^, which increases the viscosity by 37.18% compared to the base fluid. The sensitivity analysis indicated that the highest sensitivity is related to temperature and the lowest sensitivity is related to SR. Response surface method, curve fitting method, adaptive neuro-fuzzy inference system and Gaussian process regression (GPR) have been used to predict the dynamic viscosity. Based on the results, all four models can predict the dynamic viscosity. However, the GPR model has better performance than the other models.

## Introduction

Nanofluids are create of nanopowders (NPS) suspended in a base fluid (BF). During the last decade, much research has focused on rheological behavior and its applications. In the creation of nanofluid, one or more solid phases are added to the BF and augment the BF heat transfer rate. Some NPS, such as aluminum oxide, magnesium oxide and cerium oxide, are in the form of metal oxide and can be easily dispersed and suspended in liquids. Hybrid nanofluid (HNF) is a nanofluid that uses more than one type of NPS in its construction. Using two types of NPS simultaneously can create a stable combination with unique thermal properties. By the addition of NPS to the BF, its thermophysical specifications, including dynamic viscosity, are affected [1–6]. Dynamic viscosity is one of the influencing factors on pumping power and heat transfer coefficient. It affects the flow governing equations. Determining the rheological behavior as well as dynamic viscosity of hybrid nanofluids is a crucial issue in the field, and many researchers studied the viscosity variations of hybrid nanofluids under different shear rates (SRs), nanofluid volume fraction (VF) and temperature [7].

Among the studies on the Newtonian behavior of nanofluids, Soltani et al. [8] surveyed the viscosity of the MgO–MWCNT/ethylene glycol HNF in 0 < NPSVF < 1% and 30 < temperature < 60 °C. They also stated a 168% rise in the viscosity of the Newtonian HNF compared to the BF at NPSVF = 1%. They obtainable an exponential correlation to depict viscosity variations in terms of temperature (*T*) and NPSVF of HNF. Also, Asadi et al. [9] considered the rheological behavior of oil–Mg (OH)_2_/MWCNT HNF in 25 < temperature < 60 °C and 0.25 < NPSVF < 2% experimentally. Their findings showed that the HNF in all temperatures and NPSVFs have the NB. Saeedi et al. [10] explored the performance of cerium oxide–ethylene glycol (EG) nanofluid in 0.05 < NPSVF < 1.2% and a 25 < temperature < 50 °C. Their results show the Newtonian performance of the considered nanofluid. Sepyani et al. [11] testified the performance of ZnO–engine oil nanofluid in 0 < NPSVF < 1.5% and 25 < temperature < 50 °C. Aladag et al. [12] surveyed the efficacy of *T* and SR on the viscosity of carbon nanotube–H_2_O and aluminum oxide–H_2_O nanofluids. According to their results, in 2 < temperature < 10 °C, the nanofluid containing carbon nanotubes has a Newtonian behavior at high SRs. In contrast, the nanofluid including aluminum oxide has a non-Newtonian behavior. Esfe and Saedodin [13] observed a Newtonian behavior ZnO–EG nanofluid in the 0.25 < NPSVF < 5% and 25 < temperature < 50 °C.

Among the studies conducted on nanofluids with non-Newtonian behavior (NNB), Esfe et al. [14] found that the oil–copper oxide–MWCNT HNF has a non-Newtonian manner in 5 < temperature < 55 °C and 0.05 < NPSVF < 1%. Moldoveanu et al. [15] considered the rheological performance of water–aluminum oxide–titanium oxide HNF at *T* = 25 °C and 1 < NPSVF < 2%. They stated that the HNF shows NNB in the designed experiment. Liu et al. [16] calculated oil–titanium oxide–silver and oil–aluminum oxide–silver HNFs at *T* = 25 °C and 1 < NPSVF < 4%. Their findings showed that both HNFs have the NNB. Namburu et al. [17] inspected the flow features of water–EG/ copper oxide nanofluid in a laboratory study. They presented a correlation for viscosity in 0 < NPSVF < 6.12%, 35 < *T* < 50 °C. In their study, the diameter of NPS is 29 nm. Their findings described that the viscosity lessens exponentially with enhancing temperature, and viscosity rises with growing NPSVF. Kumar et al. [18] inspected the viscosity of oil–zinc–Cu hybrid nanofluid with NPSVFs of 0 to 0.5%. According to their results, the HNF viscosity rises with the enhancement in NPSVF. In a laboratory study, Eshgarf et al. [19] inspected the efficacy of *T* and NPSVF on the viscosity of water–EG–MWCNT–silicon oxide HNF. Their results displayed that the HNF has the NNB. The HNF viscosity augments by lessening temperature and rising NPSVF. Bahrami et al. [20] examined the viscosity of H_2_Or–EG–Fe–CuO HNF in 0.05 < NPSVF < 1.5% and 299 K < temperature < 323 K. According to their outcomes, this nanofluid has an NNB and the viscosity of this nanofluid lessens by growing temperature and reducing NPSVF. Afrand et al. [21] experimented the viscosity of silver–iron oxide–EG HNF in 0.037 < NPSVF < 1.2% and 25 °C < temperature < 50 °C. Their outcomes illustrated that the HNF has an NNB for NPSVF > 0.6.

Yiamsawas et al. [22] inspected the viscosity of TiO_2_ NPS/EG–H_2_O in different NPSVF and 15 < temperature < 60 °C. By extracting the correlation from the test results and comparing it with other correlations, they found that the academic correlations are not appropriate for calculating the nanofluids viscosity. Their proposed correlation was a function of BF’s viscosity, *T* and NPSVF. Cabaleiro et al. [23] experimented the viscosity of TiO_2_–EG nanofluid in 0 < NPSVF < 2.5% and 25 °C < temperature < 50 °C. By determining the viscosity at several SRs, they found that this hybrid nanofluid has an NNB. Moghadam et al. [24] surveyed the properties of graphene–glycerol nanofluids in 0.0025 < NPSVF < 0.02 and 20 < temperature < 60 °C. Based on their consequences, the viscosity of nanofluids rises by growing NPSVF and reducing temperature. They described that nanofluids are a non-Newtonian fluid. A summary of the investigations of nanofluids based on SAE50 and metal oxides is given in Tables 1 and 2. Various other researches have been done on modeling the properties of nanofluids [25–28] and investigating parameters affecting it [29–32] in order to be used in refrigeration and thermal equipment and lubrication of mechanical systems.

**Table 1:** Some studies based on SAE 50

Author	Year	NPS	NPSVF (%)	Temperature (°C)	Behavior
Esfe and Rostamian [33]	2017	TiO_2_	0.5–1	25–50	NNB
Esfe et al. [34]	2019	MWCNT–CuO	0–1	25–50	NNB
Esfe et al. [35]	2019	MWCNT–TiO_2_	0–1	25–50	NNB
Esfe et al. [7]	2022	MWCNT–Al_2_O_3_	0–1	25–55	NNB

**Table 2:** Some studies of nanofluids with metal NPS

Ref. number	Year	NPS	Base fluid	NPSVF (%)	Temperature (°C)	behavior
Abareshi [36]	2011	Fe_2_O_3_	Glycerol	0.075–1.25	30–70	NNB
Sundar et al. [37]	2012	Fe_3_O_4_	Water/EG mixture	0–1	0–50	–
Hamid et al. [38]	2015	Al_2_O_3_	Water/EG mixture	0–2	30–70	–
Esfe and Abbasian [39]	2017	MgO–MWCNT	5w50	0–1	5–55	NNB
Esfe et al. [40]	2017	MWCNT–TiO_2_	10w40	0–1	5–55	NNB
Esfe et al. [41]	2017	MWCNT–Al_2_O_3_	5w50	0–1	5–55	NNB
Esfe et al. [42]	2018	ZnO–MWCNT	10w40	0–1	5–55	NNB
Esfe et al. [43]	2018	ZrO_2_–MWCNT	10w40	0–1	5–55	NNB
Sajeeb and kumar [44]	2019	CeO_2_–CuO	Coconut oil	0–1	30–90	NNB
Esfe et al. [45]	2021	MWCNT–Al_2_O_3_	5w50	0.05–1	5–55	NNB

According to the studies presented in the literature, hybrid nanofluids containing nanomaterials such as MWCNT, TiO_2_, Al_2_O_3_, ZnO, MgO, CuO and SiO_2_ have been widely discussed in terms of rheological behavior and dynamic viscosity. However, the investigation of rheological and dynamic characteristics of hybrid nanofluids in the presence of tin oxide and cerium oxide has received less attention. Few researchers investigated the thermophysical properties of nanofluids containing tin oxide [46, 47] and cerium oxide nanopowders [10, 44, 48, 49]. Considering that the Newtonian or non-Newtonian performance of nanofluid performs a significant character in thermal and flow field, it is necessary to study its rheological behavior. According to this mater and the review done by the authors, the rheological behavior of SAE50–cerium oxide–tin oxide hybrid nanofluid is investigated, which has not been studied so far. Therefore, in the current study, the dynamic viscosity of SAE50–cerium oxide–tin oxide–oil HNF is measured in 25 < temperature < 67 °C, 0.25 < NPSVF < 1.5% and 1333 < SR < 2932.6 s^−1^. Then the efficacy of temperature, NPSVF and SR on the dynamic viscosity is investigated. CFM, RSM, ANFIS and ML are used to estimate the HNF viscosity based on the effective variables.

## Experiment

In the existing survey, a two-stage technique was used to make nanofluids. In this method, the NPS formed separately is dispersed in the BF with suitable methods. This technique is simpler and low-priced than the one-step method. Also, this technique is proper for making nanofluids with oxide NPS. To prevent clumping and adhesion of NPS, the nanofluid was first rotated using a magnetic stirrer (1200 rpm speed) for 30 min. Then, the nanofluid was subjected to the ultrasonic waves tool for 2 h. The amount of time to use the magnetic stirrer and the ultrasonic device is determined according to the number and type of nanoparticles and the user's experience. To assess the nanofluid viscosity, the viscometer CAP2000 + manufactured by the American Brookfield Company was used [50]. SAE50 engine oil generated by Behran company is employed as the BF in this research. Cerium and tin oxide nanopowders were obtained from the American Nanomaterials Research Company. Experiments have been performed for the prepared samples in 1333 < SR < 2932.6 s^−1^ and 25 < *T* < 67 °C. Table 3 shows the features of cerium oxide and tin oxide NPS. Table 4 shows the characteristics of SAE50 engine oil.

**Table 3:** Characteristics of nanopowders

Properties	Nanopowder	
	Cerium oxide (CeO_2_)	Tin oxide (SnO_2_)
Nanopowder purity	99.97%	> 99.7%
Color	Light yellow	White
Size	10–30 nm	35–55 nm
Density (*ρ*)	7.132 g/cm^3^	6.95 g/cm^3^
Specific surface area	30–50 m^2^/g	18.55 m^2^/g
Image

**Table 4:** Specifications of the base fluid

Kinematic viscosity @ 100 °C	1.8 × 10^−5^ (m^2^/s)
Viscosity Index (VI)	90
FLASH point	246 (°C)
Pour point	− 9 (°C)
Total base number (TBN)	4.1 (mgKOH/g)
Density @ 15 °C	0.906 (g/cm^−3^)
Specific heat	1900 (J/Kg K)

**Fig. 1:**
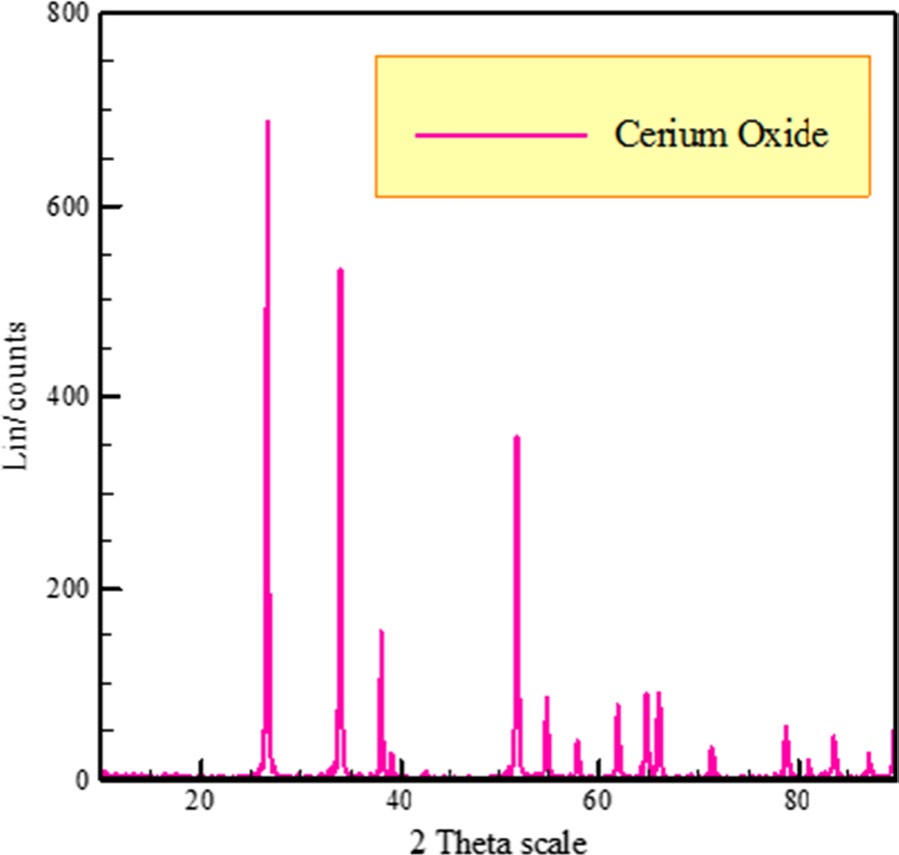
XRD of cerium oxide nanopowders

**Fig. 2:**
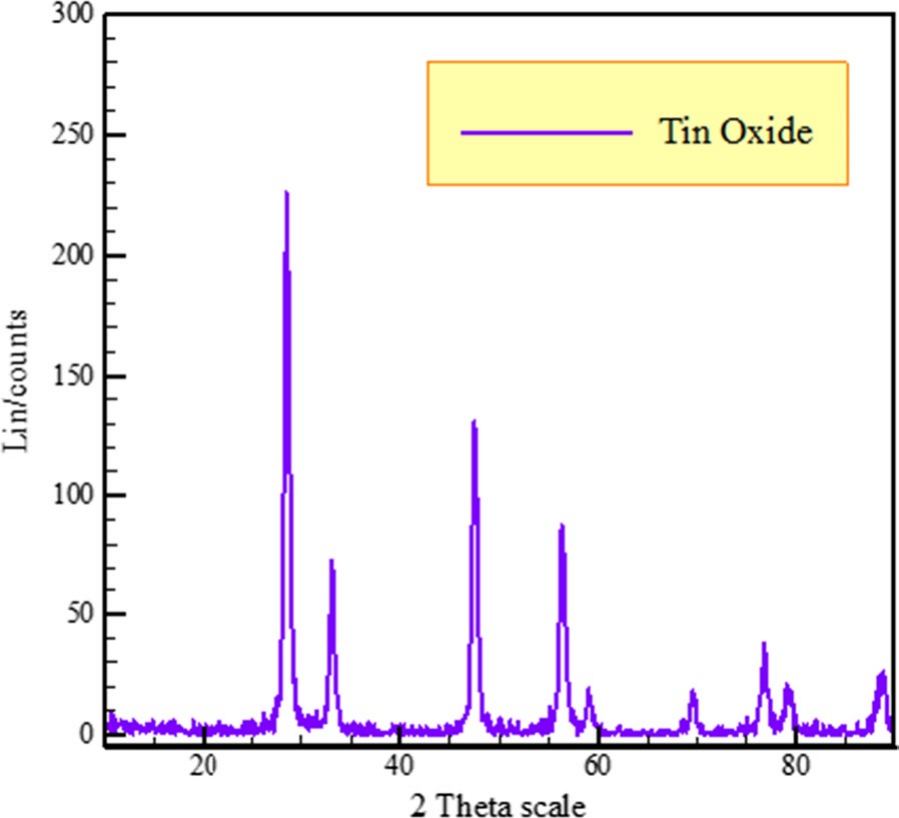
XRD of tin oxide nanopowders

The X-ray technique was employed to assess the NPS construction and size. Figures 1 and 2 show XRD images of CeO_2_ NPS and SnO_2_ NPS, respectivel. The pointed and thin peak in the XRD diagram indicates that both nanopowders of cerium oxide and tin oxide have very good crystal phase structure.

NPSVF ($\varphi$) is defined based on the mass (*w*) and density ($\rho$) of nanopowders and oil as follows:

1 $$\varphi = \frac{{\left( {\frac{w}{\rho }} \right)_{{{\text{Cerium}}\;{\text{oxide}}}} + \left( {\frac{w}{\rho }} \right)_{{{\text{Tin}}\;{\text{oxide}}}} }}{{\left( {\frac{w}{\rho }} \right)_{{{\text{Cerium}}\;{\text{oxide}}}} + \left( {\frac{w}{\rho }} \right)_{{{\text{Tin}}\;{\text{oxide}}}} + \left( {\frac{w}{\rho }} \right)_{{{\text{SAE}}50}} }} \times 100$$

**Fig. 3:**
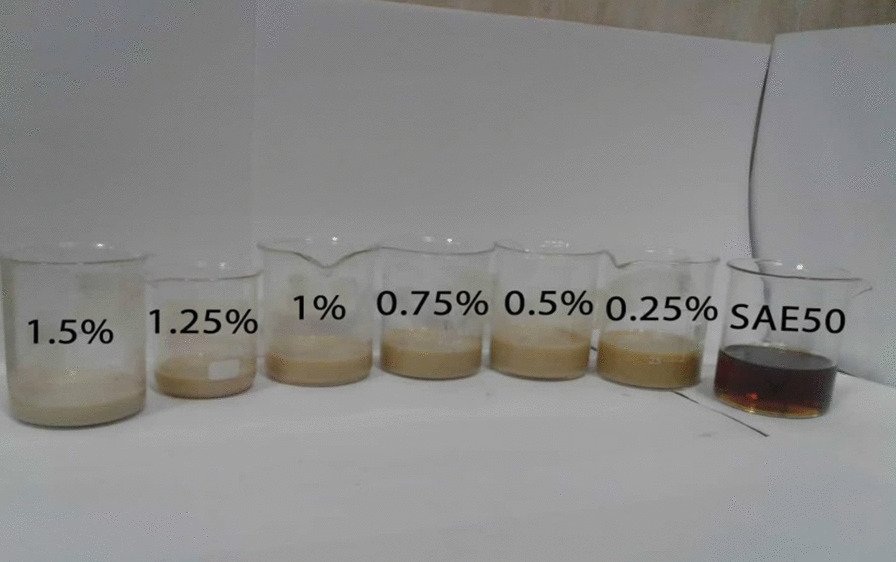
Hybrid nanofluid samples prepared respectively in various NPSVF

Hybrid nanofluid samples are shown in Fig. 3. The stability of nanofluids was checked in four weeks, and no lumpiness was observed.

## Results and Discussion

The rheological performance of SAE50 oil–tin oxide–cerium oxide hybrid nanofluid is studied in the laboratory. Nanofluids in NPSVFs (0.25 to 1.5%) were made by a two-step method. Experiments have been executed at 25 < *T* < 67 °C and 1333 < SR < 2932.6 s^−1^.

### Nanofluid Behavior

**Fig. 4:**
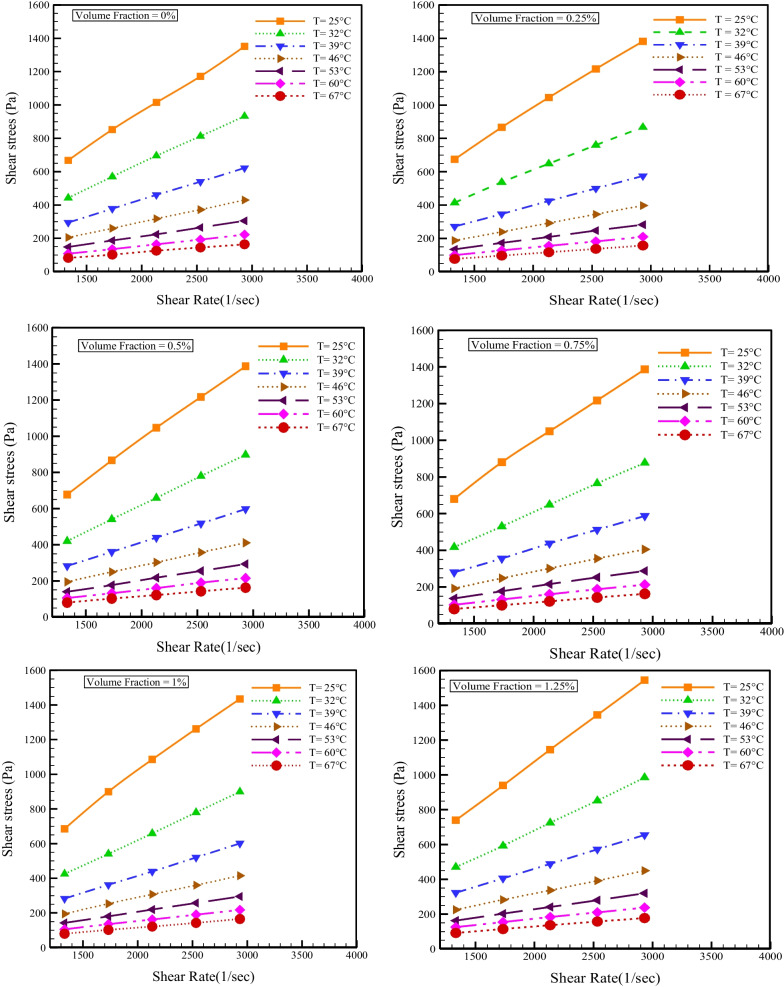

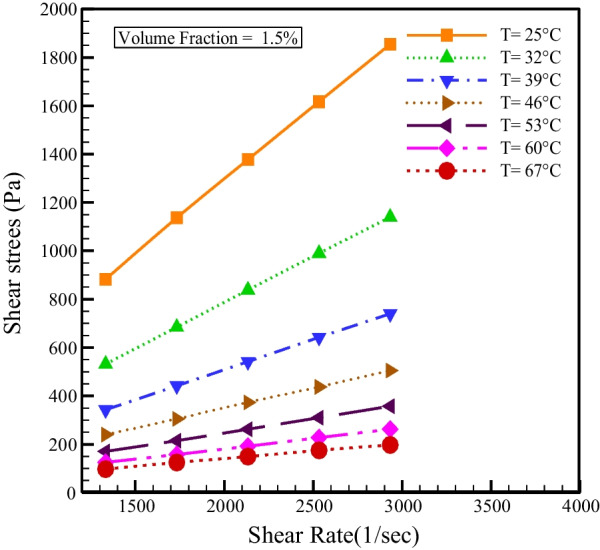
Shear stress changes according to SR for several NPSVFs and temperatures

Variations of shear tension with SR for different NPSVFs and temperatures are shown in Fig. 4. As expected, and regardless of the nanofluid's rheological behavior, shear stress increases in all NPSVFs with increasing SR because SR and shear stress are directly related in Newtonian and non-Newtonian fluids. The results show that in all NPSVFs for a constant SR, the shear stress and viscosity reduce with the increment in temperature. The decrement in $\mu$ causes a decrease in the shear stress. Also, in all the NPSVFs, the shear stress graph slope with the SR increases with decreasing temperature, which indicates the NNB of the SAE50 engine oil and the cerium oxide–tin oxide/oil hybrid nanofluid in the investigated NPSVFs.

**Fig. 5:**
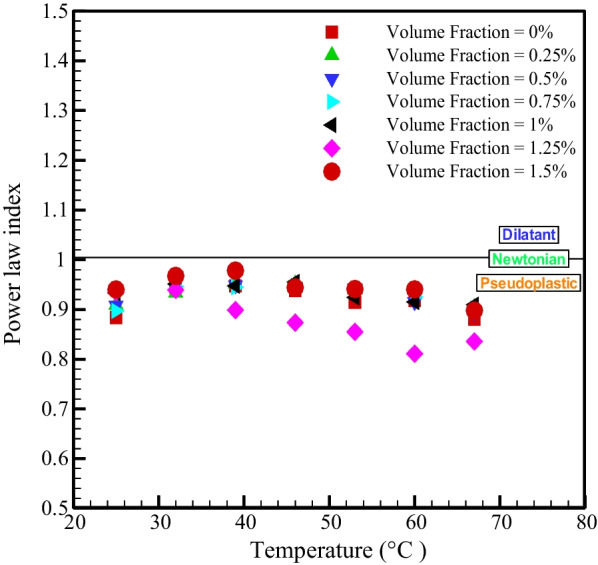
Changes of power index with temperature in different NPSVFs

**Fig. 6:**
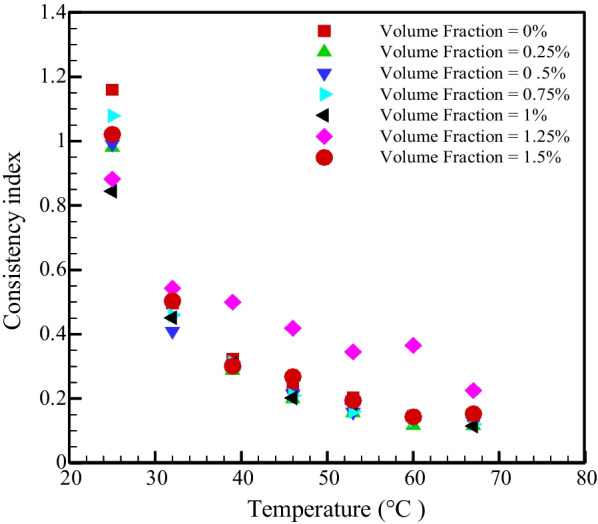
Changes of strength index with temperature for different NPSVFs

The shear tension for power-law fluid is given as follow as:

2 $$\tau = m\dot{\gamma }^{n}$$

where $$\tau , \;m,\;n\; {\text{and}}\; \dot{\gamma }$$ are the shear stress, strength index, index of flow power and SR. Figure 5 shows the changes of the power index ($$n)$$ in terms of *T* in different NPSVFs. $$n<1$$ for all temperatures and NPSVFs, which specifies the quasi-plastic behavior of the prepared nanofluid. Figure 6 shows the changes in strength index ($$m$$) according to temperature for different NPSVFs. According to Fig. 6, the strength index lessens with enhancing temperature in all NPSVFs. The viscosity is a result of the intermolecular force. With the increment in temperature, the energy level of the molecules increases and can overcome the intermolecular adhesion force.

**Fig. 7:**
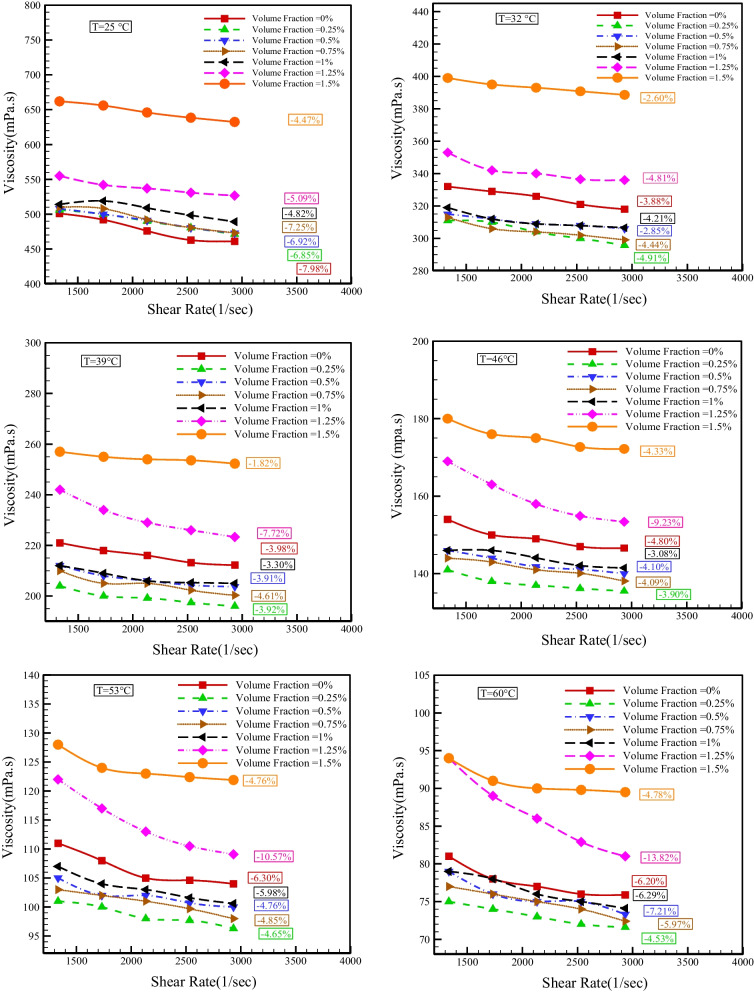

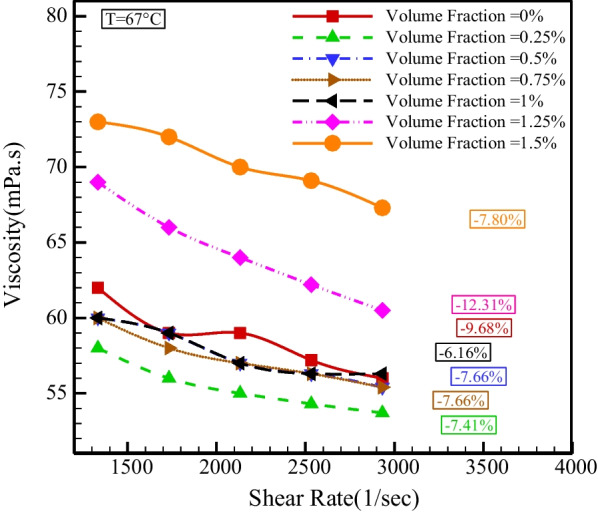
Changes in dynamic viscosity with SR at different temperatures

Figure 7 demonstrates the dynamic viscosity changes with SR at different temperatures. The consequences indicate that dynamic viscosity lessens with boosting SR, which confirms the NNB of base fluid and HNF. The lowest reduction in viscosity with an increase in SR is 1.82%, which happens at *T* = 39 °C and NPSVF = 1.5%. The most significant decrease in viscosity with an increase in the SR is 13.82%, which happens at *T* = 60 °C and NPSVF = 1.25%.

**Fig. 8:**
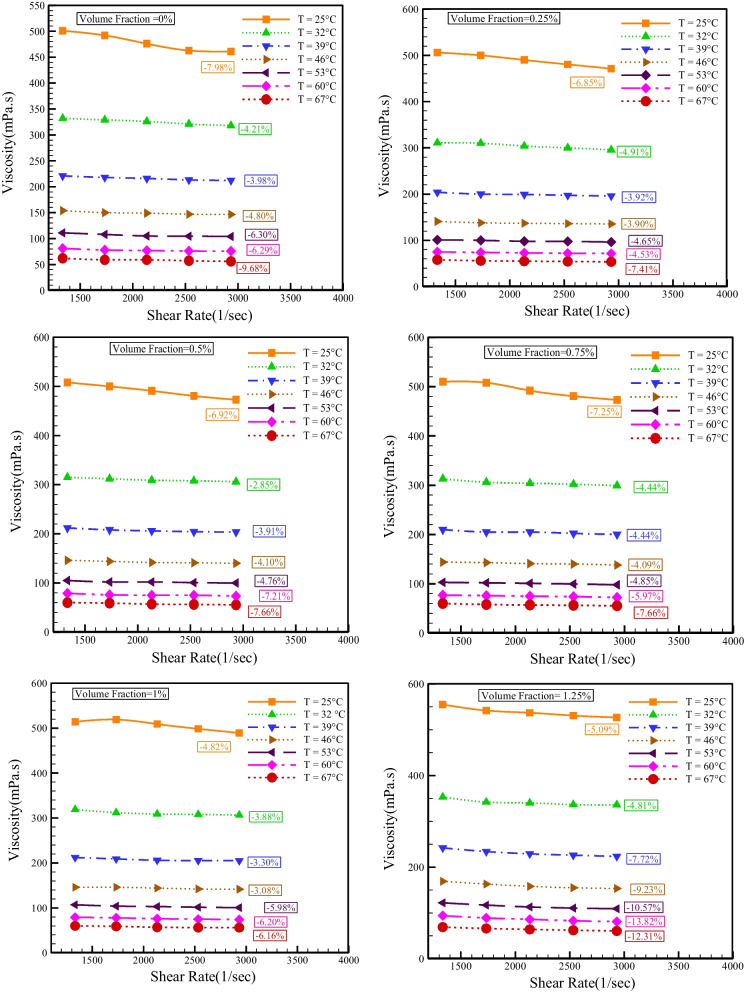

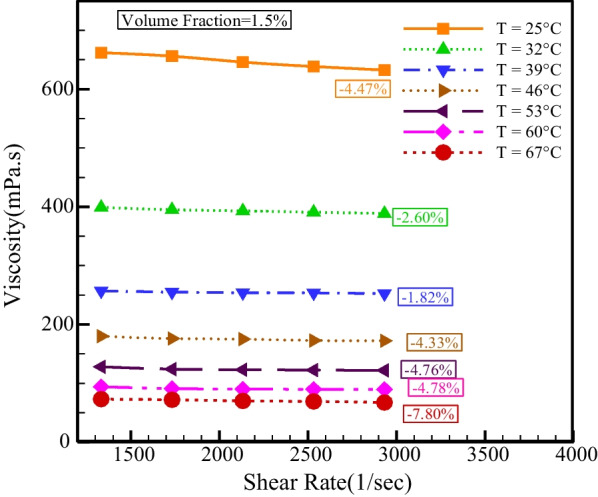
Changes in dynamic viscosity according to shear rate in different NPSVFs

Figure 8 displays the dynamic viscosity variations with SR in different NPSVFs. The outcomes specify that *µ* reduces in all cases with rising SR, which confirms the NNB of base fluid and HNF.

**Fig. 9:**
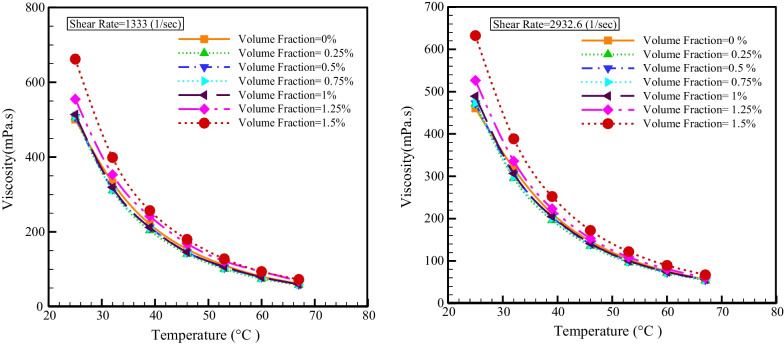
Viscosity changes with the temperature at different SRs

Figure 9 shows the viscosity changes with temperature at different SRs. Temperature is an influencing factor on viscosity, and an increment in temperature causes a decrement in the van der Waals force, which decreases the fluid's resistance to movement, so the relationship between temperature and viscosity is inverse, and in all NPSVFs, because of the augment in temperature, the viscosity decreases. As the *T* augments from 25 to 67 °C, the viscosity lessens between 87.57 and 89.36%.

**Fig. 10:**
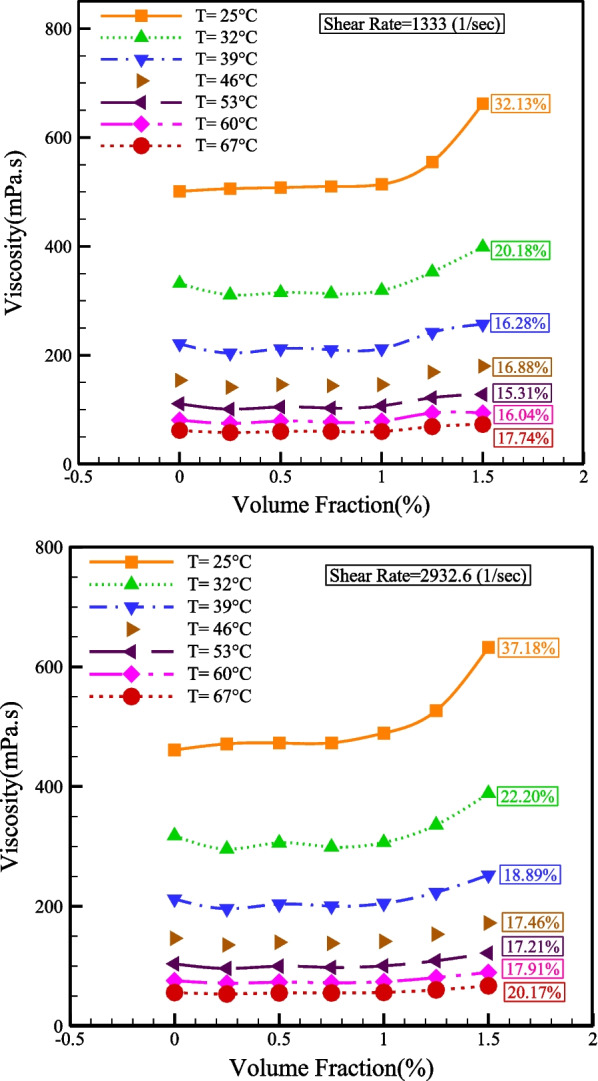
Changes in viscosity with NPSVF at different SRs

Figure 10 reveals the change of viscosity with NPSVF at different SRs. The NPSVF has a direct relationship with the viscosity because the addition of NPS increases the van der Waals force, which increases the fluid's resistance to movement, so at all temperatures, the viscosity reduces with the reduction in the NPSVF. The greatest increase in viscosity occurs with the rise in NPSVF at the minimum temperature (25 °C) so that at SRs of 1333 and 2932.6 s^−1^, the dynamic viscosity increases by 32.13% and 37.18%, respectively.

**Fig. 11:**
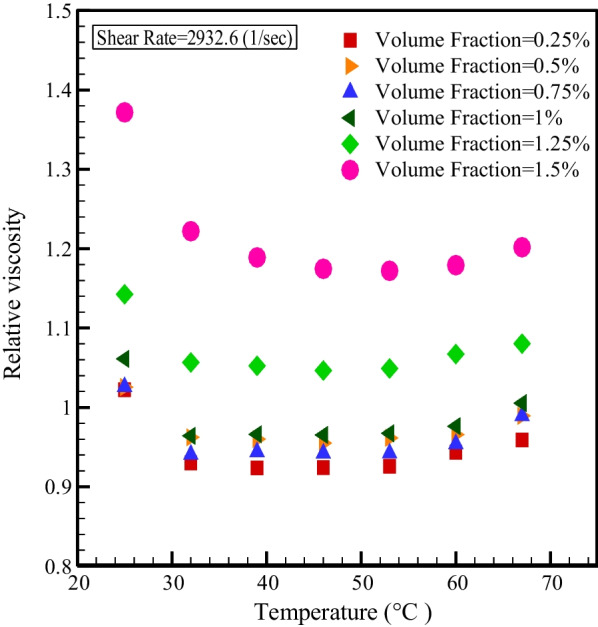
Relative viscosity changes with temperature at constant SR

**Fig. 12:**
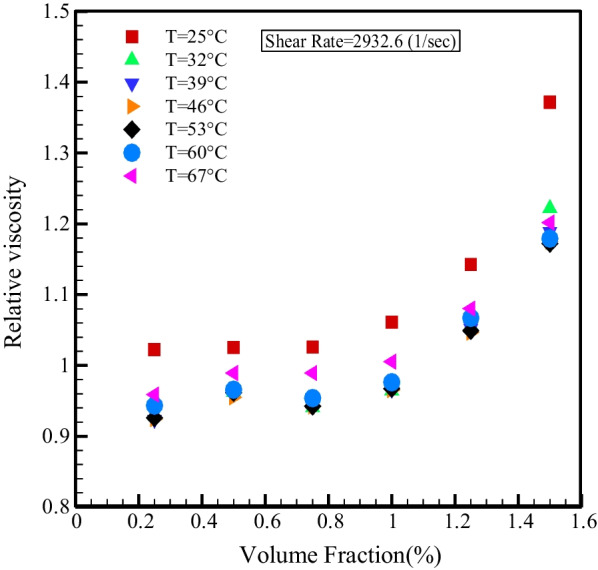
Relative viscosity change in terms of NPSVF at constant SR

Figure 11 displays the relative viscosity changes with *T* for SR = 1333 and 2932.6 s^−1^. As can be seen, in the NPSVFs of 1.25% and 1.5%, the relative viscosity is more than one at all temperatures. Still, in other NPSVFs, the relative viscosity is more than one only at *T* = 25 °C, and in other temperatures are NPSVFs smaller than unity. Figure 12 demonstrates the relative viscosity changes with NPSVF at constant SR. It is understood, at *T* = 25 °C, the relative viscosity is greater than one in all NPSVF values, but at other temperatures, the relative viscosity is greater than one only for NPSVFs greater than 1%. According to the results of Figs. 11 and 12, the highest relative viscosity arises at T = 25 °C and NPSVF = 1.5%, which increases the viscosity by 37.18% compared to the base fluid.

**Fig. 13:**
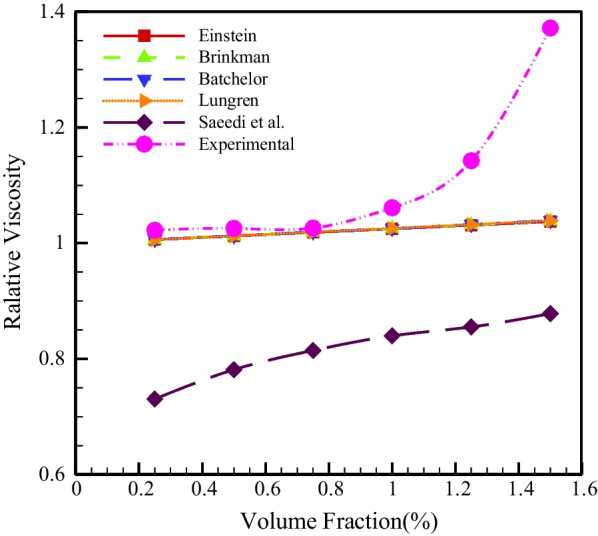
Comparison of relative viscosity with different models

Figure 13 displays the comparison of the relative viscosity at *T* = 25 °C and SR = 2932.6 s^−1^ with the models of Einstein [51], Brinkman [52], Bachelor [53], Lundgren [54] and Saeedi et al. [10]. According to Fig. 13, in NPSVF < 1%, the experimental values obtained in the present study have a slight difference from the models of Einstein [51], Brinkmann [52], Bachelor [53] and Lundgren [54], but with an increase in the NPSVF for values more than 1% of the mentioned models does not have the capability to forecast the HNF performance, and therefore, a novel model for the viscosity of this HNF should be present. Table 5 shows the proposed relationships of relative viscosity.

**Table 5:** Proposed relations of relative viscosity

Author	Correlation
Einstein [51]	$\frac{{\mathop \mu \nolimits_{{{\text{nf}}}} }}{{\mathop \mu \nolimits_{{\text{f}}} }} = (1 + 2.5\varphi )\quad (3)$
Brinkman [52]	$\frac{{\mathop \mu \nolimits_{{{\text{nf}}}} }}{{\mathop \mu \nolimits_{{\text{f}}} }} = \frac{1}{{\mathop {(1 - \varphi )}\nolimits^{2.5} }}\quad (4)$
Bachelor [53]	$\frac{{\mathop \mu \nolimits_{{{\text{nf}}}} }}{{\mathop \mu \nolimits_{{\text{f}}} }} = (1 + 2.5\varphi + 6.2\varphi^{2} )\quad (5)$
Lundgren [54]	$\frac{{\mathop \mu \nolimits_{{{\text{nf}}}} }}{{\mathop \mu \nolimits_{{\text{f}}} }} = (1 + 2.5\varphi + \frac{25}{4}\varphi^{2} )\quad (6)$
Saeedi et al. [10]	$\frac{{\mathop \mu \nolimits_{{{\text{nf}}}} }}{{\mathop \mu \nolimits_{{\text{f}}} }} = 781.4*(T^{ - 2.117} )*(\varphi^{0.2722} )) + \frac{0.05776}{{(T^{ - 0.7819} )*(\varphi^{ - 0.04009} )}} + (0.511*(\varphi^{2} )) - (0.1779*(\varphi^{3} ))\quad (7)$

### Developing Model to Estimate $\mu$

In this section, using four different methods, the dynamic viscosity of the SAE50 oil–SnO_2_–CeO_2_ hybrid nanofluid is predicted. These four methods are CFM, RSM, ANFIS and GPR.

#### Curve Fitting Method (CFM)

To compute the $\mu$ of SnO_2_–CeO_2_–SAE50–oil HNF using the CFM, the following equation is presented:

8 $$\mu_{{{\text{nf}}}} = a + b\phi + cT + {\text{d}}\gamma + e\phi^{2} + fT^{2} + g\phi^{3} + hT^{3} + iT\phi + j\gamma \phi + kT\gamma$$

In the above equation, *R*^2^ = 0.9933 and the proposed model has 11 constants which are tabulated in Table 6. *R*^2^ is the parameter to evaluate the model's accuracy.

**Table 6:** Constants in Eq. (8)

Constants	Value	Constants	Value
*a*	1970	*g*	68.9
*b*	165	*h*	− 0.00882
*c*	− 92.6	*i*	− 2.09
*d*	− 0.0212	*j*	− 0.00055
*e*	− 119	*k*	0.000316
*f*	1.55		

#### RSM Method

Statistical analysis (SA) was employed to estimate the $\mu$ of cerium oxide–tin oxide SAE50 engine oil HNF by RSM. Laboratory data have been used as historical data for modeling. The input data to this model are NPSVF, *T* and SR. The output variable is $\mu$. Tables 7 and 8 show the input variables and feature response of this model, respectively.

**Table 7:** Input variables of RSM model

Factor	Name	Units	Type	Sub type	Minimum	Maximum	Coded Low	Coded High	Mean	Std. Dev
A	Volume fraction	%	Numeric	Continuous	0.2500	1.50	− 1 ↔ 0.25	+ 1 ↔ 1.50	0.8750	0.4280
B	Temperature	°C	Numeric	Continuous	25.00	67.00	− 1 ↔ 25.00	+ 1 ↔ 67.00	46.00	14.03
C	Shear rate	1/s	Numeric	Continuous	1333.00	2932.60	− 1 ↔ 1333.00	+ 1 ↔ 2932.60	2132.80	566.90

**Table 8:** Response feature of the RSM model

Response	Name	Units	Observations	Minimum	Maximum	Mean	Std. Dev	Ratio	Transform	Model
*R*1	Dynamic viscosity	mPa s	210.00	53.7	662	209.88	157.67	12.33	Power	Quadratic

Table 9 provides the SA of various models. The complexity indicates the model's number of terms. As can be seen, the quadratic function has the very good accuracy and moderate complexity and accordingly was used as the optimal model. If the cubic model is used, the complexity is doubled compared to the quadratic model, but the accuracy increases by 0.0003, so the quadratic model is selected as the optimal model.

**Table 9:** SA of different model

Source	Sequential *p*-value	Adjusted *R*^2^	Predicted *R*^2^	Complexity
2FI	< 10^–4^	0.9964	0. 9963	7
Quadratic	< 10^–4^	0.9990	0. 9990	10
Cubic	< 10^–4^	0.9993	0.9992	20
Quartic	< 10^–4^	0.9996	0.9995	35
Fifth	< 10^–4^	0.9998	0.9997	56

The variance analysis (VAAN) for the proposed model (based on the quadratic model) is shown in Table 10. The results indicate the validity of this model. The fit statistics are tabulated in Table 11. In this analysis, the R^2^ coefficient is the degree of agreement between the data predicted by the model and the laboratory data and is equal to 0.9990. This coefficient highlights the degree of fit of model data in the range of experimental data and shows the value of estimated model data for data outside the variety of experimental data. If Adeq. Precision < 4, it means that the signal-to-noise ratio is desirable [55]. In this model, Adeq. Precision is 496.4019.

**Table 10:** VAAN for the quadratic model

Source	Sum of squares	*df*	Mean square	*F*-value	*p*-value	
Model	0.5214	9	0.0579	24,005.19	< 0.0001	
A—volume fraction	0.0059	1	0.0059	2458.37	< 0.0001	Significant
*B*—*T*	0.5134	1	0.5134	2.128E + 05	< 0.0001	
C—SR	0.0005	1	0.0005	197.44	< 0.0001	
AB	0.0001	1	0.0001	57.97	< 0.0001	
AC	1.687E−06	1	1.687E−06	0.6989	0.4042	
BC	0.0001	1	0.0001	28.04	< 0.0001	
A^2^	0.0012	1	0.0012	507.27	< 0.0001	
*B*^2^	0.0001	1	0.0001	36.43	< 0.0001	
*C*^2^	8.324E−06	1	8.324E−06	3.45	0.0647	
Residual	0.0005	200	2.413E−06			
Cor total	0.5219	209

**Table 11:** Fit statistics

Std. Dev	0.0016	*R*^2^	0.9991
Mean	0.2715	Adjusted *R*^2^	0.9990
C.V. %	0.5722	Predicted *R*^2^	0.9990
		Adeq Precision	496.4019

**Fig. 14:**
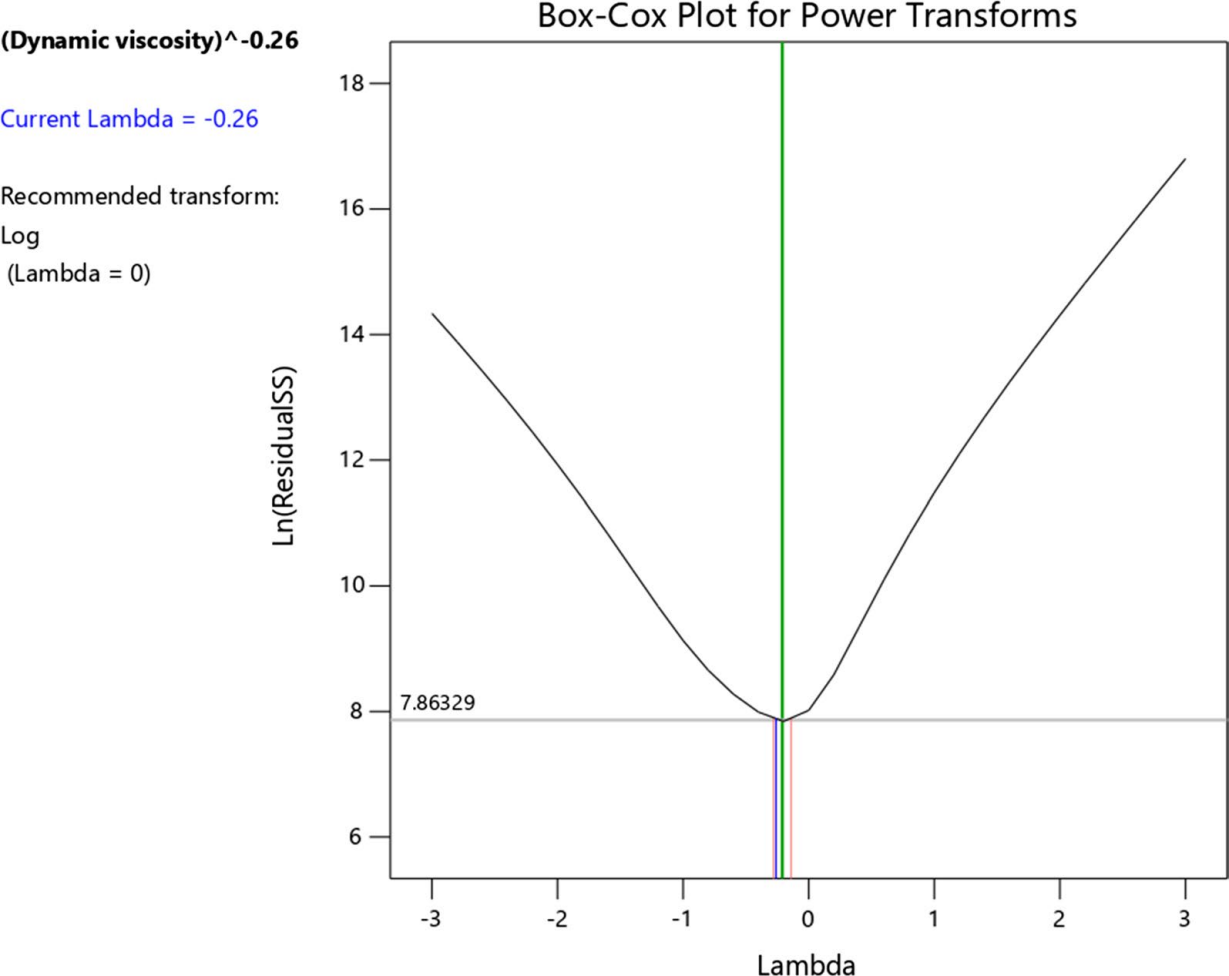
Box–Cox plot to specify the revised transform function

The Box–Cox chart based on the software is illustrated in Fig. 14. The transform function to normalize data is expressed as $y^{\prime} = (y)^{ - 0.26}$. The dynamic viscosity equation extracted from RSM is exhibited in Eq. (9). The constant coefficients of Eq. (9) are presented in Table 12. Considering the simplicity and accuracy of the proposed model from the RSM, it can be said that Eq. (9) is more appropriate for calculating the viscosity of cerium oxide–tin oxide–oil hybrid nanofluid than Eq. (8). The proposed correlations in the present work can be applied for various applications, including numerical studies [56–64], nanolubricants [65–68], enclosures [69, 70], permeable surfaces [71, 72], microchannels [73–76], heat pipes [77], heat exchangers [78–80], heat sinks [81–83], cooling [84] and the automotive industry [85–88].

9 $$\mu_{{{\text{nf}}}}^{ - 0.26} = \alpha_{0} + \alpha_{1} \phi + \alpha_{2} T + \alpha_{3} \dot{\gamma } + \alpha_{4} \phi T + \alpha_{5} \phi \dot{\gamma } + \alpha_{6} T\dot{\gamma } + \alpha_{7} \phi^{2} + \alpha_{8} T^{2} + \alpha_{9} \dot{\gamma }^{2}$$

**Table 12:** Constants in the suggested Eq. (9)

Constants	Value	Constants	Value
$\alpha_{0}$	+ 0.096955	$\alpha_{5}$	+ 3.71149E−07
$\alpha_{1}$	+ 0.020142	$\alpha_{6}$	+ 7.16927E−08
$\alpha_{2}$	+ 0.003849	$\alpha_{7}$	− 0.015487
$\alpha_{3}$	+ 2.21442E−06	$\alpha_{8}$	− 3.81169E−06
$\alpha_{4}$	− 0.000137	$\alpha_{9}$	− 7.44006E−10

**Fig. 15:**
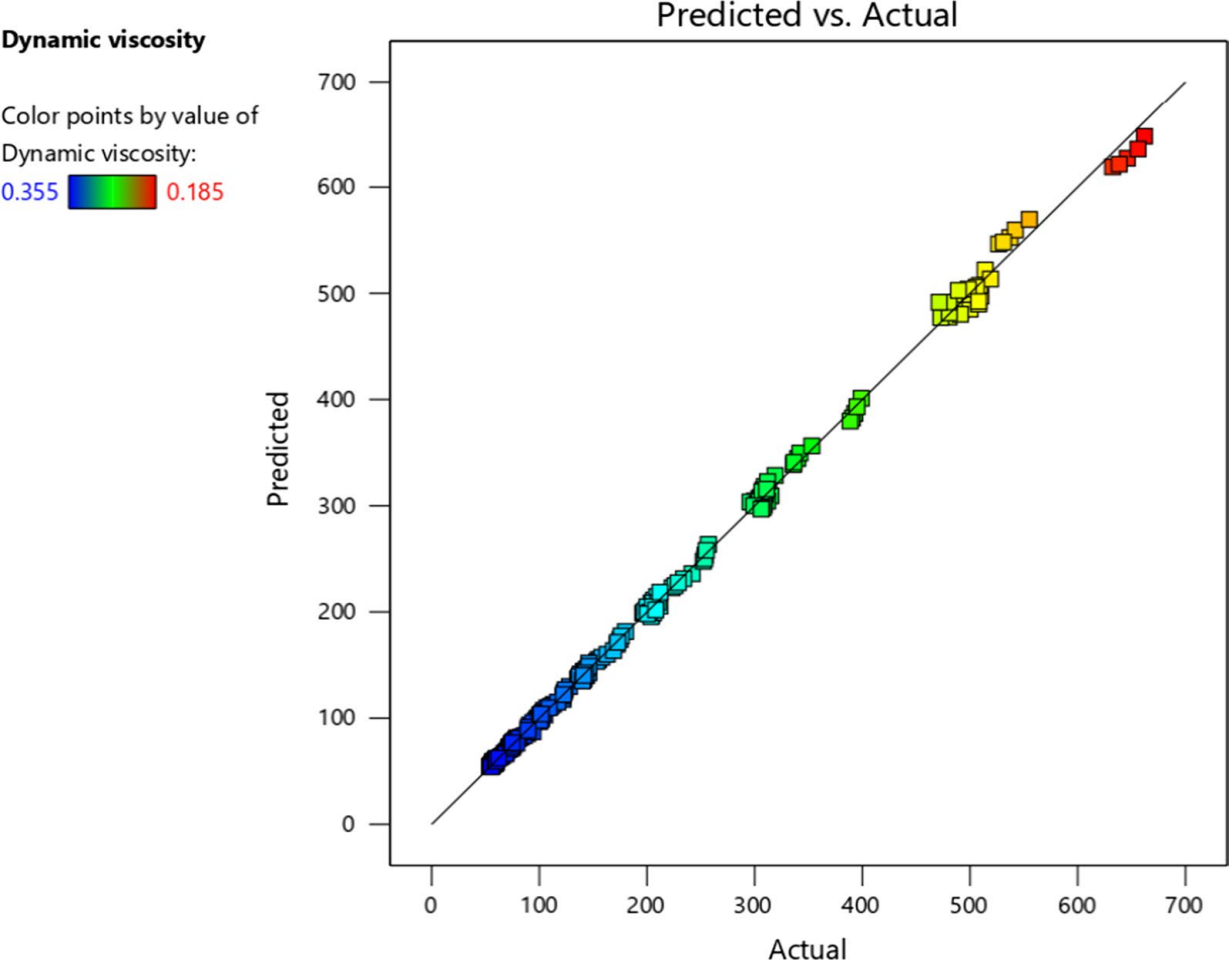
Comparison between estimated and experimental data

**Fig. 16:**
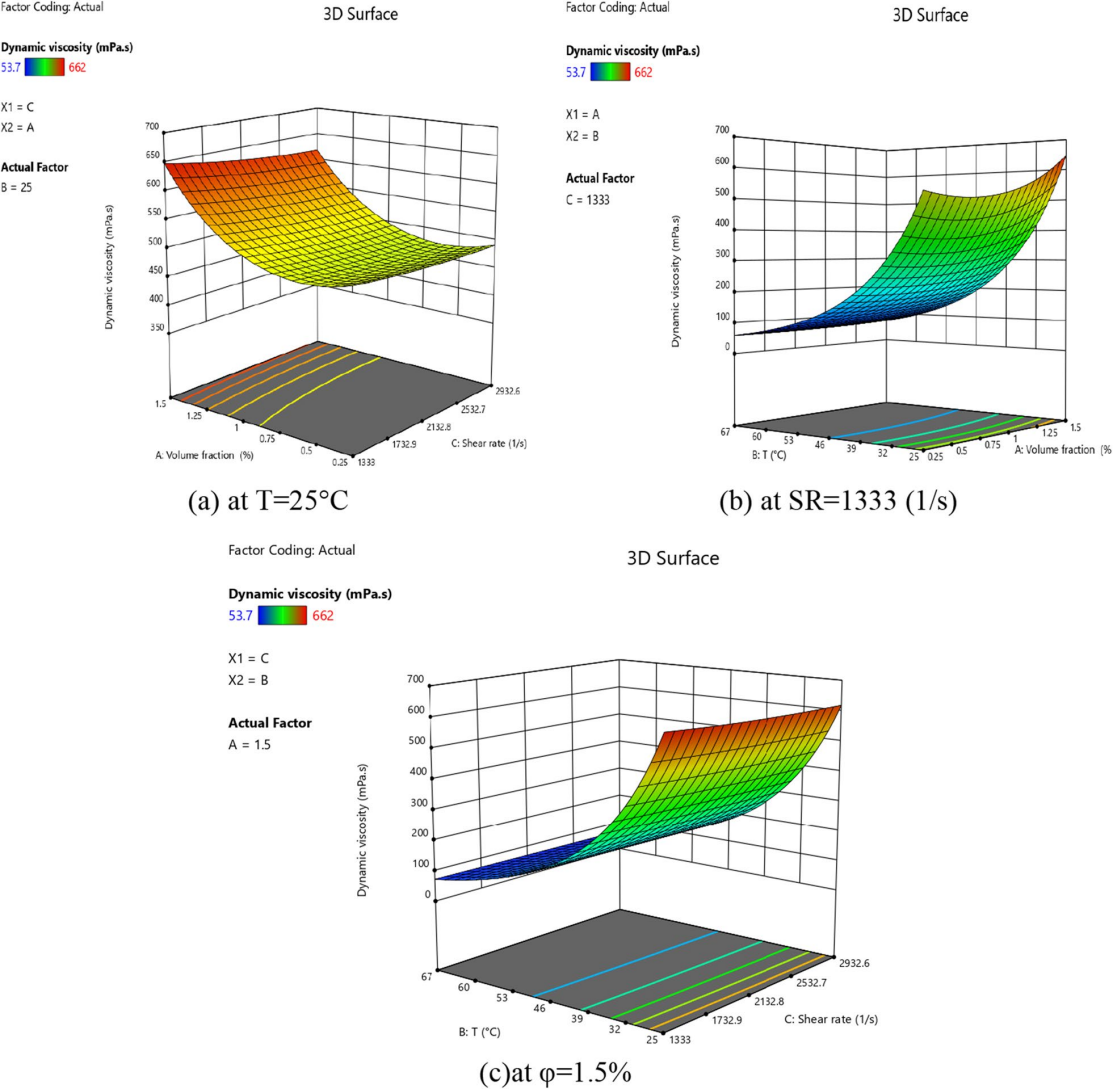
Interaction within **a** SR and NPSVF, **b**) *T* and NPSVF, and **c** *T* and SR on viscosity by RSM

Figure 15 reveals the regression graph. There is a good agreement between the estimated and the actual data. The 3D surface charts of the demonstration accomplished from the statistical examination are plotted in Fig. 16. As well, the efficacy of the T, NPSVF and SR on the model is plotted.

#### Machine Learning (ML): Adaptive Neuro-Fuzzy Inference System (ANFIS)

Machine learning, as one of the new fields of computer science, has attracted the attention of researchers in various fields of engineering in the last few decades. One of the goals of this science is to investigate and invent algorithms based on which the computer can perform learning and prediction based on a limited set of data [89]. In supervised learning, ML is based on sets of labeled observations, output for inputs. Modeling systems with common mathematical tools such as differential equations are not suitable and efficient for complex systems with uncertainty. On the other hand, fuzzy systems by using a set of fuzzy rules can model the qualitative aspects of human knowledge and reasoning processes without the use of detailed quantitative analysis [90]. Fuzzy neural networks are obtained by combining fuzzy structures with artificial neural networks, which are used to identify systems and predict time series and various other cases. The structure of ANFIS is the result of the integration of adaptive neural networks and fuzzy logic; by applying the hybrid learning process, its parameters can be adjusted to model systems based on the existing input–output data [90]. It combines the advantage of using adaptive neural network and fuzzy logic.

The structure of the ANFIS model consists of five layers as follows: The first layer is the input nodes; in this layer, the degree of membership of the input nodes (the degree of belonging of each input) to different fuzzy intervals is determined by the user using the membership function. Modeling operations are performed in the second to fourth layers. By multiplying the input values to each node, the weight of each rule in the second layer is obtained. In the third layer, the relative weights of the rules are calculated. In the fourth layer, each node has a node function and is connected to all inputs and a node in the third layer. The last layer is the output of the network, whose purpose is to summarize all the output of the rules [91, 92].

In the training phase, by modifying the parameters of the degree of membership based on the acceptable error, the output values become closer to the real values. Common training methods are error back propagation and hybrid methods. In the error backpropagation method, using the gradient descent algorithm, the error value is propagated to the inputs and the parameters are corrected. In the hybrid method, the combination of gradient descent and least squares error is used. The random selection of data is one of the points that should be considered in training and testing the ANFIS network [91]. In the present study, MATLAB software was used for modeling. In this modeling, 75% of laboratory data have been used for training and 25% for testing. In total, 265 experimental data are used for modeling.

**Fig. 17:**
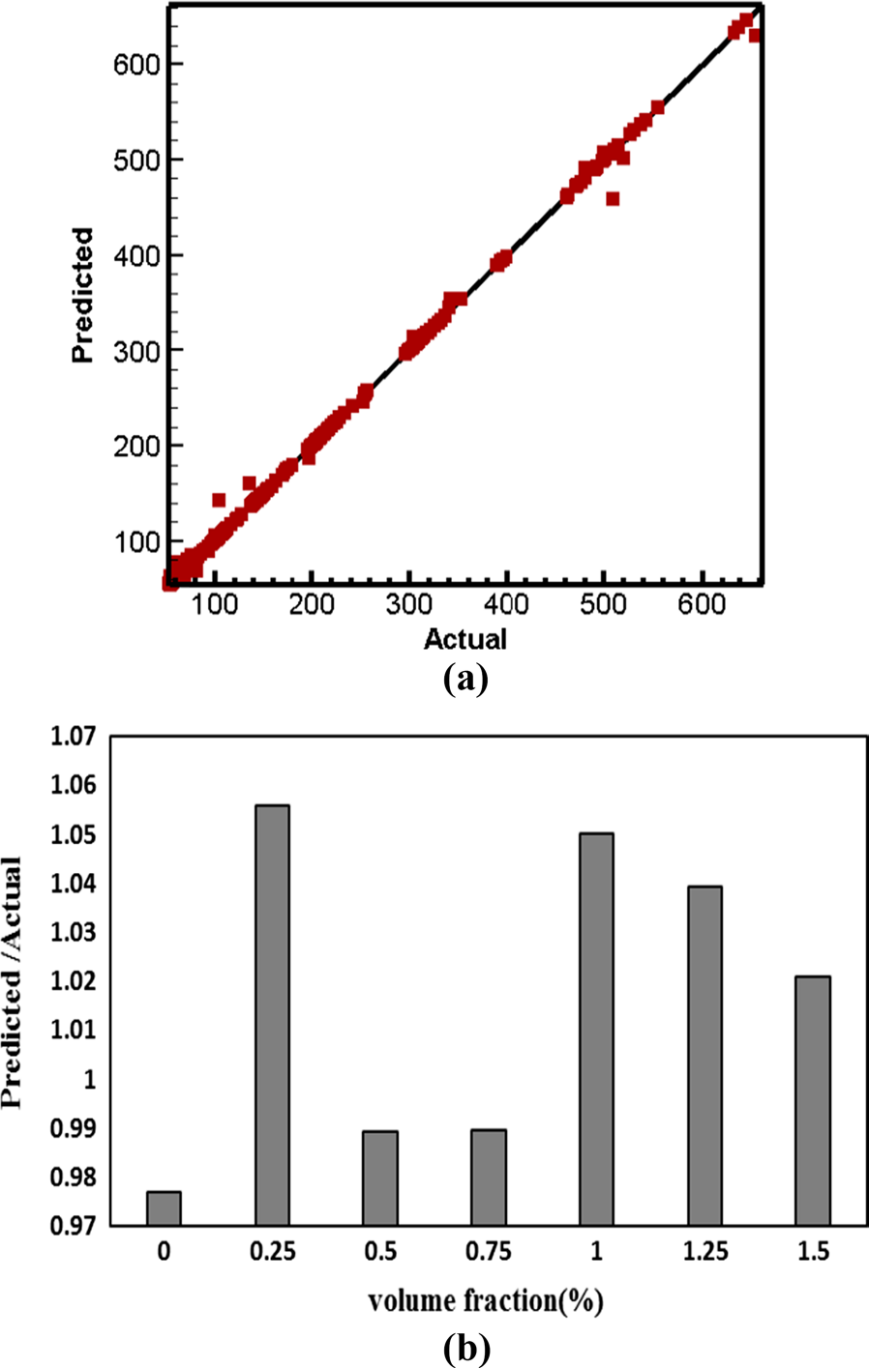
**a** Comparison of ANFIS model with experimental data and **b** ratio of the predicted viscosity to the actual state in *T* = 46 °C and SR = 1739.2 s^−1^in terms of NPSVF

**Fig. 18:**
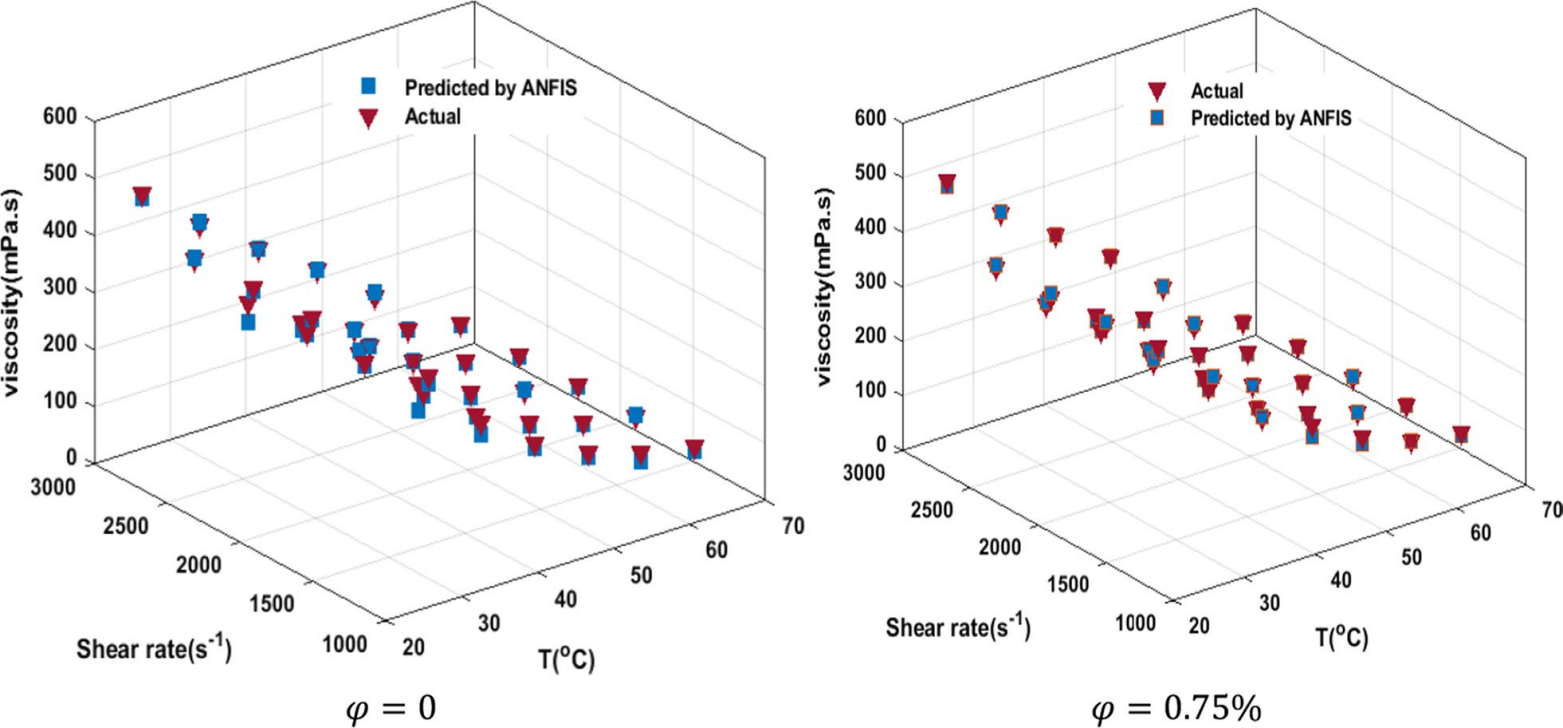
Comparison of estimated data by ANFIS model with experimental data

The outcome of the viscosity prediction using the ANFIS is shown in Fig. 17a. The prediction of the ANFIS model offers a strong correlation (*R*^2^ = 0.9945) with the viscosity investigation of the current study. The errors (mPa s) were found to be 16.76 (RMSE), 28.81 (MSE). For example, the ratio of the predicted viscosity to the actual state in $T = 46\;^{ \circ } {\text{C}}\;{\text{and}}\;{\text{SR}} = 1739.2\;{\text{s}}^{ - 1}$ in terms of NPSVF is shown in Fig. 17b. Figure 18 compares the predicted and actual data in $\varphi =$ 0 and 0.75%. The results indicate the ability of the ANFIS model to predict viscosity.

#### Machine Learning: Gaussian Process Regression (GPR)

One of the important issues in supervised learning is regression [93], in which despite the limited number of observations for a function, we want to obtain a model for it in order to estimate its value in points that have not been observed. GPR is a nonparametric Bayesian approach to regression that makes waves in the field of machine learning. GPR has several advantages, works well on small data sets and has the ability to provide a measure of uncertainty in predictions. Unlike many supervised ML algorithms that learn exact values for each parameter in a function, the Bayesian method infers a probability distribution over all possible values.

Gaussian process (GP) is a set of random variables, a limited number of which are integrated with Gaussian distributions. Each process has a common Gaussian distribution. Gaussian distribution is actually a distribution between random variables, while the Gaussian process represents a distribution between functions. GPR analysis provides a way to classify data based on the structures embedded in them. In the GP, a function called distribution function f is defined. In this process, f is a mapping from the input space X to the space R. GPR algorithm models are based on the assumption that the set observations should carry information about each other. Gaussian processes are a way to view a priori directly on the function space. Gaussian distribution is on vectors, while GP is on functions. As a result, GP models do not require any validation process due to prior knowledge of functional dependencies and data for generalization, and GPR models are able to understand the predictive distribution corresponding to the test input [93].

**Fig. 19:**
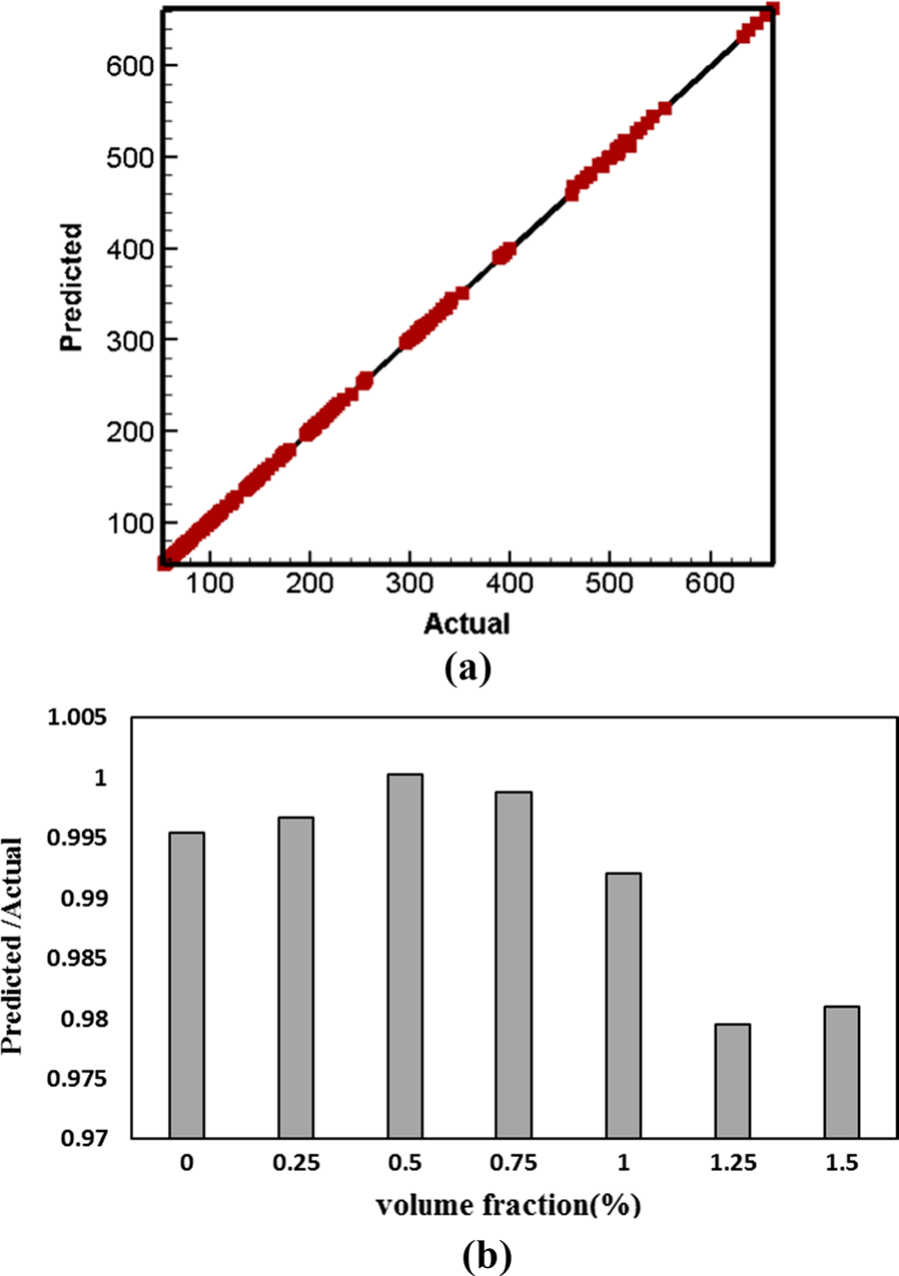
**a** Comparison of GPR model results with experimental data, and **b** ratio of the predicted viscosity to the actual state in *T* = 46 °C and SR = 1739.2 s^−1^in terms of NPSVF

**Fig. 20:**
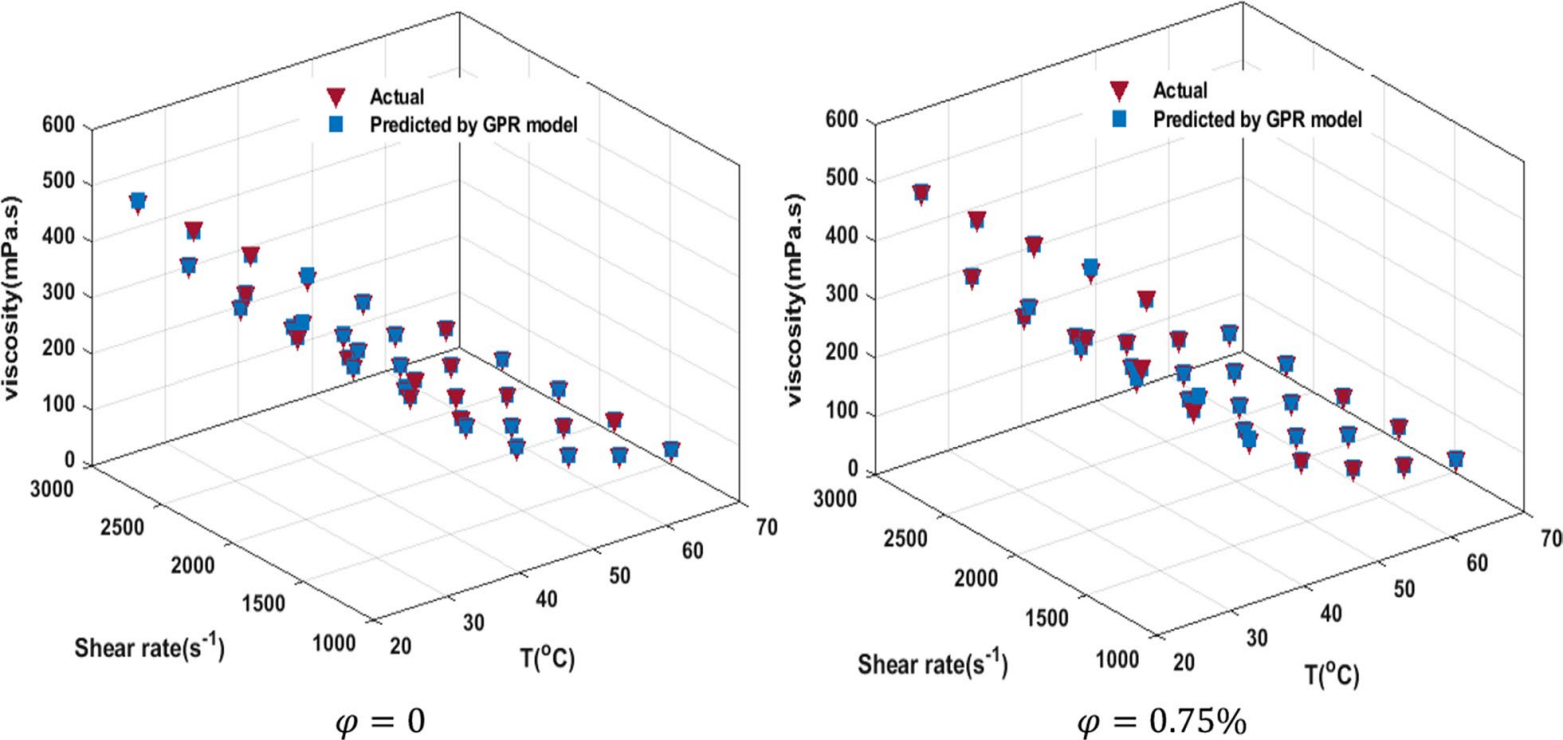
Comparison of estimated data by GPR model with experimental data

The outcome of the viscosity prediction using GPR based on the supervised ML is shown in Fig. 19a. The prediction of the GPR model offers a strong correlation (*R*^2^ = 1) with the viscosity investigation of the current study. Also, the kernel function is Nonisotropic Matern 3/2 and the Basis function is linear. The model is optimized based on the minimum MSE by Bayesian optimization. The training time for this analysis was 4.52 s, and the errors (mPa s) were found to be 2.147 (RMSE), 4.61 (MSE), and 1.56 (MAE). For example, the ratio of the predicted viscosity to the actual state in $T = 46\;^{ \circ } {\text{C}}\;{\text{and}}\;{\text{SR}} = 1739.2\;{\text{s}}^{ - 1}$ in terms of NPSVF is shown in Fig. 19b. The evaluation between actual and predicted data is done and illustrated in Fig. 20. The results specify the capability and accuracy of the GPR model in predicting viscosity. Among the models used in this research, the GPR model has been able to estimate the data with good accuracy and has been successful in comparison with other models.

### Sensitivity Analysis

Sensitivity analysis is used to determine the sensitivity of ${\mu }_{\mathrm{nf}}$ to changes in SR, *T* and NPSVF. For this purpose, sensitivity is defined as:

10 $${\text{Sensitivity}}\; \left( \% \right) = \left( {\frac{{\mu_{{{\text{nf,}}\;{\text{After}}\;{\text{change}}}} - \mu_{{{\text{nf,}}\;{\text{Base}}\;{\text{condition}}}} }}{{\mu_{{{\text{nf,}}\;{\text{Base}}\;{\text{condition}}}} }}} \right) \times 100$$

**Fig. 21:**
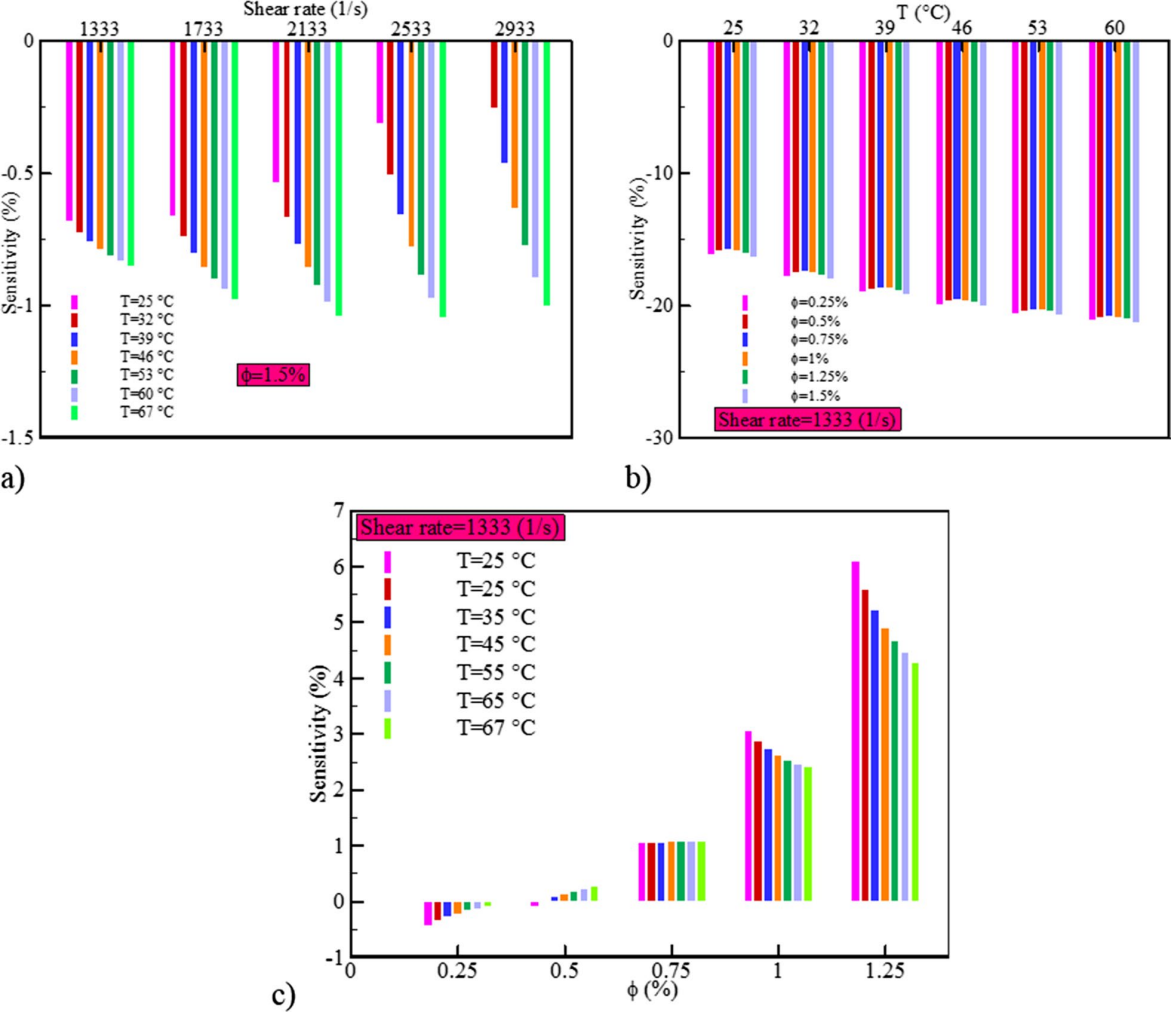
Sensitivity analysis diagram for the cerium oxide–tin oxide/SAE50 hybrid nanofluid

Sensitivity analysis is a criterion that displays the dependency of dependent variable (${\mu }_{\mathrm{nf}}$) to a certain change in each of independent variables (*T*, NPSVF and SR). The sensitivity behavior to changes of + 10% by SR, T and NPSVF is plotted in Fig. 21. The results specify that at $\varphi =1.5\%$, with the increase in the SR (Fig. 21a), the changes in sensitivity do not have a significant and uniform trend, and the maximum value of sensitivity is about 1%. Also, the average sensitivity increases with increasing temperature (Fig. 21b), so that the sensitivity value at *T* = 25 °C and 60 °C is about 16% and 21%, respectively. At a constant SR, the sensitivity increases with the increase in the NPSVF (Fig. 21c), so that for the NPSVF of 0.25%, 0.5% and 0.75%, the maximum sensitivity is about 1%, but for NPSVF = 1% and 1.25%, it is 3% and 6%, respectively.

## Conclusion

In this exploration, the viscosity of tin oxide–cerium oxide hybrid nanofluid was examined in the temperature (25–67 °C), NPSVF (0.1–5%) and SR (1333–2932.6 s^−1^). The results specified:

- At all considered states, the nanofluid has a non-Newtonian pseudo-plastic performance.
- At a constant temperature for all NPSVFs, as the SR increases and the temperature decreases, the shear stress increases.
- The dynamic viscosity decreases with increasing SR and temperature and decreasing NPSVF. It can also be seen that viscosity is very sensitive to temperature changes compared to other parameters. The highest relative viscosity occurs at *T* = 25 °C and NPSVF = 1.5%, which shows that the nanofluid viscosity has augmented by 37.18%.
- The coefficients of determination of the four models: CFM, RSM, ANFIS and ML are 0.9933, 0.9990, 0.9945 and 1, respectively. Therefore, the GPR model extracted from the ML is more accurate than other models.

## Data Availability

All data analyzed during this study are included in this published article.

## List of symbols

*m*: Strength index (Pa s^n^)
*n*: Index of flow power
*T*: Temperature (°C)
*w*: Mass (kg)

## Greek

$\varphi$: Nanopowder volume fraction
$$\dot{\gamma }$$: Shear rate (s^−^^1^)
$\tau$: Shear stress (Pa)
$$\rho$$: Density (kg m^−^^3^)
$\mu_{{{\text{nf}}}}$: Nanofluid dynamic viscosity (Pa s)
$\mu_{{\text{f}}}$: Fluid dynamic viscosity (Pa s)

## Abbreviations

ANFIS: Adaptive neuro-fuzzy inference system
BF: Base fluid
CFM: Curve fitting method
GPR: Gaussian process regression
HNF: Hybrid nanofluid
NB: Newtonian behavior
NNB: Non-Newtonian behavior
NPS: Nanopowders
NPSVF: Nanopowder volume fraction
RSM: Response surface method
SR: Shear rate
ML: Machine learning
EG: Ethylene glycol
VAAN: Variance analysis
RMSE: Root mean square error
MSE: Mean square error
